# Supplementary material for: From sequence to enzyme mechanism using multi-label machine learning
Source: BMC Bioinformatics. 2014 May 19;15:150. doi: 10.1186/1471-2105-15-150 (PMC4229970; doi:10.1186/1471-2105-15-150)
Supplement: Additional file 2 — Java code of ml2db. Additional file ml2db_code.tar.gz contains the Java source code to run the multi-label machine learning experiments and save the results to database. The code’s Javadoc is included. [file 1471-2105-15-150-S2.zip › additional file 2/ml2db/utils/doc/edu/cornell/lassp/houle/RngPack/package-frame.html]

edu.cornell.lassp.houle.RngPack


# edu.cornell.lassp.houle.RngPack

## Classes

- RandomApp
- RandomElement
- RandomJava
- RandomSeedable
- RandomShuffle
- Ranecu
- Ranlux
- Ranmar
